# Prediction of the incubation period for COVID-19 and future virus disease outbreaks

**DOI:** 10.1186/s12915-020-00919-9

**Published:** 2020-11-30

**Authors:** Ayal B. Gussow, Noam Auslander, Yuri I. Wolf, Eugene V. Koonin

**Affiliations:** National Center for Biotechnology Information, National Library of Medicine, National Institutes of Health, Bethesda, MD 20894 USA

**Keywords:** SARS-CoV-2, COVID-19, Coronavirus, Incubation period, Respiratory infections, Pandemic, Respiratory disease, Machine learning

## Abstract

**Background:**

A crucial factor in mitigating respiratory viral outbreaks is early determination of the duration of the incubation period and, accordingly, the required quarantine time for potentially exposed individuals. At the time of the COVID-19 pandemic, optimization of quarantine regimes becomes paramount for public health, societal well-being, and global economy. However, biological factors that determine the duration of the virus incubation period remain poorly understood.

**Results:**

We demonstrate a strong positive correlation between the length of the incubation period and disease severity for a wide range of human pathogenic viruses. Using a machine learning approach, we develop a predictive model that accurately estimates, solely from several virus genome features, in particular, the number of protein-coding genes and the GC content, the incubation time ranges for diverse human pathogenic RNA viruses including SARS-CoV-2. The predictive approach described here can directly help in establishing the appropriate quarantine durations and thus facilitate controlling future outbreaks.

**Conclusions:**

The length of the incubation period in viral diseases strongly correlates with disease severity, emphasizing the biological and epidemiological importance of the incubation period. Perhaps, surprisingly, incubation times of pathogenic RNA viruses can be accurately predicted solely from generic features of virus genomes. Elucidation of the biological underpinnings of the connections between these features and disease progression can be expected to reveal key aspects of virus pathogenesis.

## Background

The recent outbreak of the novel SARS-CoV-2 coronavirus and the resulting COVID-19 disease has led to an unprecedented worldwide emergency [1]. Per the World Health Organization (WHO) recommendations, numerous countries have taken severe preventive measures to combat and stem the spread of the virus. A key effective measure recommended by the WHO in viral outbreaks is enforcing a period of quarantine on individuals that are suspected to have come in contact with the causative agent until they are proven clean of infection [2, 3]. The length of the quarantine depends on the time from virus exposure to the emergence of symptoms, i.e., the incubation period. The duration of the incubation period is specific to the causative virus [4]. Underestimation of the incubation time could lead to infected individuals being prematurely released from quarantine and spreading the disease, whereas overestimation can have a debilitating economic impact and cause detrimental psychological effects [5]. Therefore, knowledge of the range and upper limit of a virus incubation period is crucial to effectively combat and prevent outbreaks while minimizing the negative consequences of the quarantine.

The length of the incubation period varies both across and within virus families [4]. Investigation of different incubation periods within a single virus species has shown that in some cases, a longer incubation period corresponds to less severe symptoms [6, 7] whereas others demonstrate the opposite trend [8]. However, to our knowledge, the association between the incubation period and severity across different human viral diseases has not been studied systematically. Further, genomic features (if any) that correlate with the incubation time are currently unknown. There is therefore a vital need for a comprehensive investigation of viral incubation periods and for methods that predict the incubation periods of emerging viruses. If such methods are developed, they can be deployed in future virus outbreaks for early, accurate inference of the incubation period and immediate implementation of optimized quarantining interventions that will mitigate the spread of the virus while minimizing the negative societal impact [9].

Here, we comprehensively assess the incubation periods of different viruses that cause human diseases. We find that, when comparing across different virus species, a longer virus incubation period is significantly associated with a more severe disease presentation. This trend is maintained within and across virus families, regardless of the affected tissue, and is especially strong among coronaviruses, and overall, for human respiratory diseases. For an in-depth examination and construction of a predictive model, we narrowed our focus to respiratory, non-segmented, single-strand RNA (ssRNA) viruses and analyzed different genomic characteristics of these viruses. We identified features that are predictive of the incubation time and are generalizable across virus families. Based on these features, we developed an elastic net regression model that predicts virus incubation periods. We extensively validated the robustness of this model and the selected features for the prediction of the incubation time across diverse viruses and virus families, to enable accurate early estimation of the incubation period for future outbreaks.

## Results

### Association between incubation period and disease severity

**Fig. 1:**
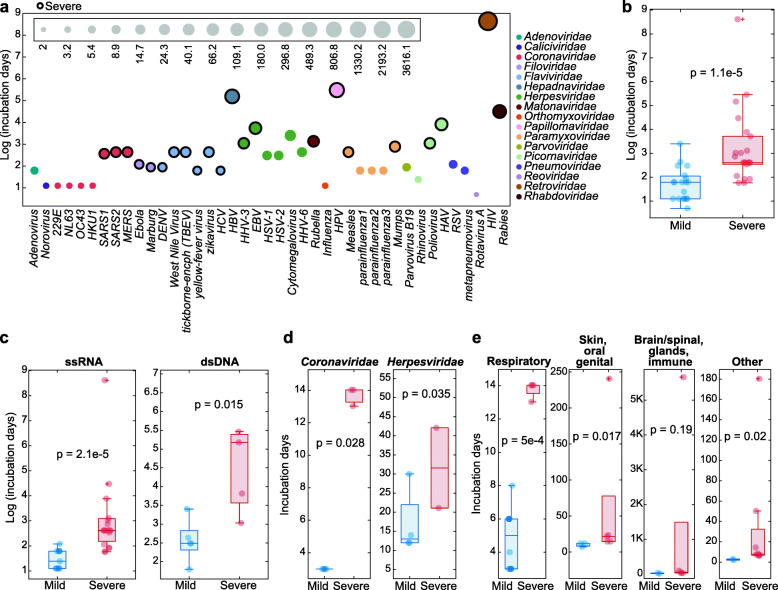
The incubation periods of viruses causing severe and mild human diseases. **a** Incubation periods (*y*-axis, log-scaled) of 41 human pathogenic viruses. The circle size corresponds to the length of the incubation period (the size scale is provided in the inset), and the colors correspond to the virus family. Severe diseases are indicated with a black border. **b** Comparison of the incubation periods (*y*-axis, log-scaled) between viruses causing mild (blue) vs. severe (red) human diseases, considering all 41 viruses collected. **c** Comparison of the incubation periods (*y*-axis, log-scaled) between viruses causing mild (blue) vs. severe (red) human disease, for ssRNA and dsDNA viruses. **d** Comparison of the incubation periods (*y*-axis, log-scaled) between viruses causing mild (blue) vs. severe (red) human disease, for large virus families. **e** Comparison of the incubation periods (*y*-axis, log-scaled) between viruses causing mild (blue) vs. severe (red) human disease, for diseases associated with distinct tissues. All indicated *P values* are for the one sided rank-sum test

**Fig. 2:**
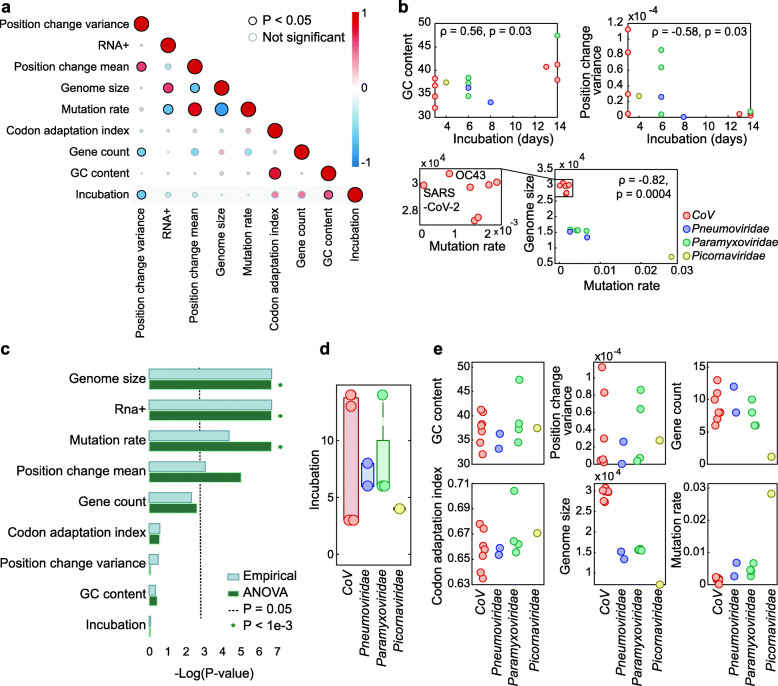
Genomic features of ssRNA viruses causing respiratory infections. **a** Pairwise correlation matrix across all features. A description of feature construction is given in the “Methods” section. Each circle indicates Spearman’s correlation coefficient (*ρ*) between two features. The colors represent the rank-correlation coefficients (red indicates positive correlation and blue indicates negative correlation), and the circle sizes correspond to significance (*p* value), where significant correlations (*p* value < 0.05) are circled in black. **b** Scatter plots illustrating the relationships between features across four virus families. **c** Estimation of the features’ association with the virus family, based on *p* values (−log-scaled) from two tests applied (see the “Methods” section for details). The cutoff (*p* value = 0.05) is indicated with a dashed line. Lower values correspond to features that are not significantly associated with a virus family. **d** Boxplot and overlaid dot plot of the incubation periods across viral families. **e** Dot plots of different features across virus families

We first curated the information on incubation periods for viral human diseases, where such data were available (41 viruses, Additional file 1: Table 1). To gain further insight into the relevance of the viral incubation periods to human disease, we investigated the relationship between viral incubation periods and disease severity. We classified diseases as severe or mild, based on the severity of the symptoms and associated death rate, following the descriptions of health organizations where applicable (see the “Methods” section for details). We found that, although the incubation periods vary substantially for the set of viruses collected, both within and across families, the viruses that cause severe disease presentations tend to have significantly longer incubation periods (Fig. 1a, *p* value 1.1e−5). This trend is strongest when considering all 41 viruses and diseases (Fig. 1b), but holds for both ssRNA and double-strand DNA (dsDNA) viruses separately (Fig. 2c). Furthermore, this trend is significant when considering the two largest viral families in this set, *Coronaviridae* and *Herpesviridae* (Fig. 1d), and among diseases associated with a particular tissue type (Fig. 1e). The biology behind the relationship between incubation period duration and disease severity warrants further exploration, but the significant association identified here between these two disease-related variables stresses the importance of the incubation period duration for both fundamental understanding of the diseases and practical health care issues.

### Prediction of incubation time from genomic features

We next sought to develop a model that would facilitate prediction of incubation periods solely from genomic features. To our knowledge, this is the first attempt to predict incubation periods from virus genomes. Given the considerable variability observed in the incubation periods among viruses that infect different tissues and those with different genome types (Fig. 1c, e), we sought to focus on a relatively homogenous subset of virus families, to minimize the risk of confounding the prediction with features of no direct relevance. To this end, we focused on non-segmented ssRNA viruses that cause respiratory infections, which is the largest group of human viruses that are relatively homogenous biologically but show considerable variation in their incubation periods (Additional file 1: Table 1). Although the predictor is built on a limited set of 14 viruses, there is a sufficient number of genomes to train the model (*n* = 3604 strains). Given that the quarantine time is defined as the upper limit of the virus incubation time, we extracted the upper estimates of the incubation periods for all viruses in the analyzed set (Additional file 1: Table S2, see the “Methods” section for details).

To train a model with the dataset in hand, we required a set of features that potentially could be predictive of the incubation times. Given that this is the first attempt, to the best of our knowledge, to identify such features, we were not aware of any established mechanistic relationships between characteristics of virus genomes and incubation periods. Thus, we selected features that are easily derived from the viral genomes and could be relevant for the incubation period (see the “Methods” section for details). We constructed 8 such features (Fig. 2a), based on the complete genome nucleotide sequences and within-population genome alignments of all sequenced strains of each virus (Additional files 1 and 2, see the “Methods” section for details). In addition to these 8 features, we also assessed CpG islands as a potential feature, because some viruses, such as hepatitis B virus (HBV), have been shown to contain varied distributions of CpG islands across different strains [10]. Furthermore, CpG avoidance has been reported for diverse RNA viruses including coronaviruses [11, 12], possibly as a result of selection against recognition by the Zinc-finger Antiviral Protein (ZAP) which binds to CpG motifs [13]. However, the extent of CpG suppression appears to be largely uniform among RNA viruses [11]. Moreover, using standard criteria [14], we did not find any CpG islands in our virus set, making it unlikely that derivations of this feature would help incubation period prediction beyond the impact of the GC content. Analysis of the pairwise associations between the 8 features (Fig. 2a) confirmed some previously reported connections, such as the negative correlation between genome length and mutation rate [15] and the positive correlation between GC content and codon adaptation index [16] (CAI) (Fig. 2 a,b). Strikingly, our findings indicate that the mutation rate of SARS-CoV-2 is substantially lower than those of other human coronaviruses (CoV), including its closest human-infecting relative, SARS-CoV (with an average of 1.4e−3 and 7.8e−5 transitions per branch point per nucleotide for SARS-CoV and SARS-CoV-2, respectively; Fig. 2b, see the “Methods” section for details). Preliminary reports on SARS-CoV-2 genome evolution indicate a similar trend [17].

We then sought to select features to be used for a predictive model of the incubation time. To avoid confounding the model with features that are primarily driven by virus family, we formally quantified whether a given feature is significantly associated with the family identity. To this end, we applied two complementary approaches, namely, analysis of variance (ANOVA) and an empirical, non-parametric test, to estimate, for each feature, whether it varies more across virus families than within each family (see the “Methods” section for details). The results obtained with the two approaches were equivalent, demonstrating that half of the considered features varied more between families than within families, and therefore might confound the model (Fig. 2c). We denote such features family-specific. By contrast, the incubation time was not significantly associated with virus family (Fig. 2d), supporting selection of features that are not family-specific to train a model; we denote such features family-generic. Four other features were found to be family-generic: GC content of the virus genome, variance of the number of different nucleotides observed per position in the alignment of the virus strains, number of protein-coding genes in the virus genome, and CAI of the virus coding sequence (Fig. 2e). Thus, these four features were included in the model.

**Fig. 3:**
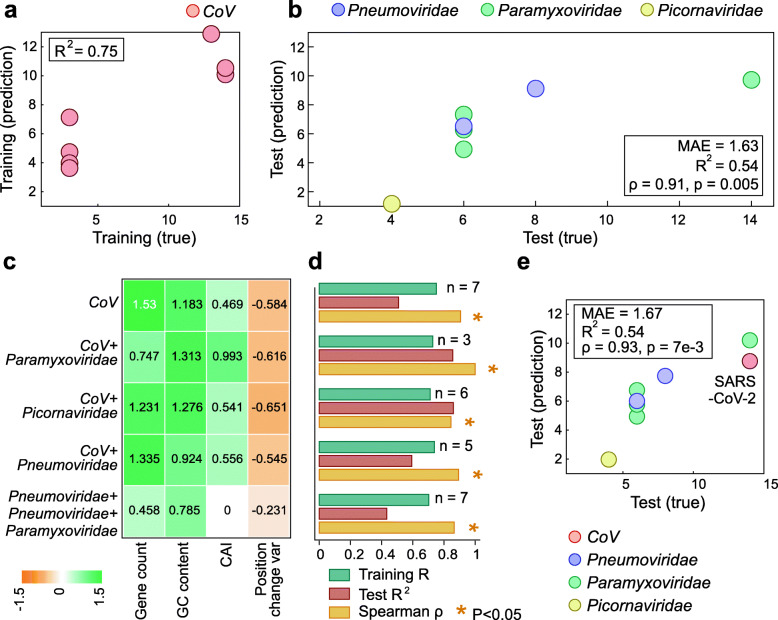
Assessment of the elastic net model for virus incubation time prediction. **a** A scatter plot of the incubation periods of the CoV training set compared to the model predictions, with the model *R*^2^ in the upper left corner. **b** A scatter plot of the incubation periods of the test set compared to the model predictions. The box in the bottom right corner contains Spearman’s *ρ* between the predictions and the true values, the *p* value of the Spearman’s *ρ*, the model *R*^2^, and the mean absolute error (MAE). **c** A heatmap of the coefficients of each feature using different training sets. **d** A bar plot of the model performance metrics using different subsets of training and testing data, with the number of samples in the testing data for each subset indicated. **e** A scatter plot of the incubation periods of the test set compared to the model predictions when SARS-CoV-2 is left out of training. The box in the top left corner shows the model’s performance metrics

Next, we divided the analyzed dataset into training and test sets. To maintain a large, diverse, and independent test set that spans multiple virus families, we selected the 7 human-infecting viruses of the family *Coronaviridae* as the training set. By training on a single viral family, we allow for a test set with the largest possible number of families, encompassing high genomic diversity and allowing for a comprehensive evaluation of the model. Moreover, coronaviruses include viruses with both high and low incubation periods, providing a good representation of the range of incubation period values. Thus, we trained an elastic net model on the 7 human-infecting viruses of the family *Coronaviridae* (Fig. 3a), using the four family-generic features. We found that this model, which was trained on a single viral family, generalized well to viruses from the three other families (Fig. 3b). The test mean absolute error was 1.63 days (Fig. 3b), attesting to a close estimation of the upper limit of the incubation time in an independent data set. Moreover, the model predictions strongly correlated with the ranks of the assigned incubation periods in the test set (Spearman’s *ρ* = 0.91, *p* value = 0.005). Specifically, for the virus with the longest known incubation period, measles, the longest incubation time, 9.7 days, was predicted. Although measles was assigned an upper limit incubation period of 14 days in our data, the majority of the available reports are indeed in the range of 9–12 days [18]. The second longest incubation period was also correctly assigned to respiratory syncytial virus (RSV), with a prediction of 9.1 days, closely approximating an assigned period of 8 days in our data. For parainfluenza viruses 1–3, the model predicted 7.3, 4.9, and 6.2 days, respectively, closely approximating the assigned 6 days. Metapneumovirus was similarly accurately predicted to have a 6.5-day incubation period, within half a day of its assigned 6 days. Finally, the shortest incubation time predicted was correctly assigned to rhinovirus, with a prediction of a 1.2-day incubation period. Although rhinovirus was assigned a 4-day incubation period in our data, most of the cases show symptoms within 1 day [19].

Exploration of the model indicated that the strongest predictive features were the number of protein-coding genes and GC content, with higher values in either feature corresponding to a longer incubation time (Fig. 3c). Elucidation of the mechanisms behind these associations will require extensive experimental work. A straightforward, even if, likely, over-simplified explanation could be that the larger number of genes to be translated by the virus lengthens its replication cycle, under the assumption that the number of translation initiation events and/or subgenomic RNAs that need to be transcribed are rate-limiting factors in virus reproduction. Similarly, a higher GC content leads to the formation of stable secondary structures in the virus RNA, with higher kinetic barriers that the ribosome then needs to disrupt during translation, resulting in longer translation times [20]. Thus, one possible explanation for the association between the number of protein-coding genes and the GC content and longer incubation periods is that the longer cumulative translation time extends the replication cycle and, consequently, the incubation period. Alternatively or additionally, extra genes could contribute to more complex interactions of the virus with the host organism, resulting in longer incubation times. In particular, the highly virulent coronaviruses with long characteristic incubation periods encode additional, accessory proteins compared to low virulence viruses that have shorter incubation times [21, 22]. The accessory genes are dispensable for virus reproduction in cell culture and have been implicated in virus-host interactions [23]. Some of these additional genes encode proteins containing distinct immunoglobulin-like domains, which is compatible with roles in interactions with the immune system of the host [24].

To assess the robustness of the selected features, we tested models trained with different partitioning of the data into train and test sets. We found that these changes did not significantly change the performance of the models, further attesting to the robustness of the signal obtained using the four family-generic features (Fig. 3d). By contrast, a model trained with family-specific features does not generalize to the test set, and one trained using a mixture of family-generic and family-specific features disregards the latter by nullifying their coefficients (Additional file 1: Figure S1), further demonstrating the efficacy of relying on family-generic features only. The coefficients assigned to the family-generic features did not vary substantially across different training sets, confirming that the method is not particularly sensitive to the data used for training (Fig. 3c). Nevertheless, the high performance of the model that is trained exclusively on CoV seems to suggest that this virus family provides a good representation of the dependencies of the incubation period on genomic features, and/or that training on a single family is preferable given the small dataset and the possibility of confounding effects.

To evaluate the utility of our model, we examined how this method would have performed during the early stages of the current COVID-19 pandemic. To this end, we removed SARS-CoV-2 from the training data and trained the model on the remaining 6 CoV only, with the caveat that this training set is poorly balanced as it contains only 2 viruses with incubation times longer than 3 days and, therefore, might underestimate when predicting viruses with long incubation times. The incubation period of SARS-CoV-2 is still being determined, with the recommended quarantine time conservatively set at 14 days. Recent reports indicate that the vast majority of symptomatic patients develop symptoms within 10.5 days, generally, within 5 days [25, 26]. Despite having trained the model on an imbalanced training set biased towards shorter incubation periods, the model predicts an incubation period of 8.8 days for SARS-CoV-2, correctly placing SARS-CoV-2 in the upper range of incubation periods and predicting an incubation period duration during which current research indicates the majority of symptomatic patients will have shown symptoms. Moreover, a recent meta-analysis that examined reported SARS-CoV-2 incubation periods across 18 studies concluded that the quarantine time should be shortened to 7 days [27]. Clearly, the estimate provided by the model could have been useful in mitigating the COVID-19 pandemic (Fig. 3e; similar analysis for the other CoV is provided in Additional file 1: Figure S2).

**Fig. 4:**
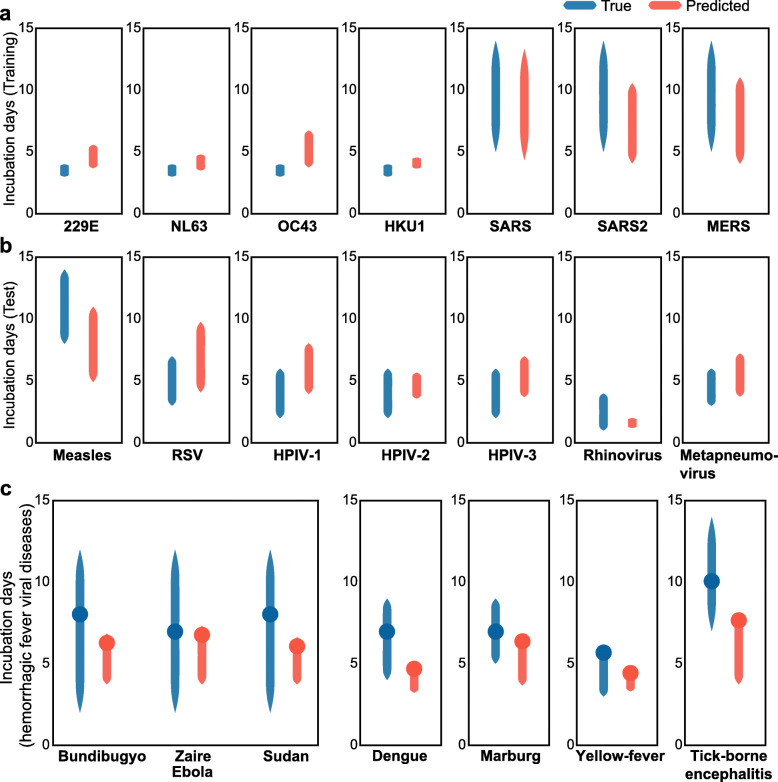
Interval evaluation for the predicted incubation period. **a** True (blue) and predicted (orange) intervals for the coronaviruses in the training set. **b** True (blue) and predicted (orange) intervals for the viruses in the test set. **c** True (blue) and predicted (orange) intervals for the viruses causing hemorrhagic fever. The blue (true) circles denote the mode value of the reported incubations periods, and the orange (predicted) circles denote the incubation period predicted by the original model (as in Fig. 3)

We further expanded the approach to facilitate an interval prediction, to provide for the prediction of the full range of incubation periods for a novel virus. Given that there is no consensus as to how to define standard errors or confidence intervals for elastic net regression models [28–30], we introduce an empirical evaluation of lower and upper ranges of the interval of the incubation period (see the “Methods” section for details). We find that the model is predictive of these intervals, in both the training set (Fig. 4a) and the test set (Fig. 4b, permutation test *p* value < 1e−3). On average, the predicted range captures 54% of the true range for viruses in the test set; at least, 30% of the true range is covered for all viruses in the test set, and at least 50% is covered in five of the seven viruses. The average absolute deviation is 1.6 days from the lower incubation range and 1.8 days from the upper incubation range.

Given the success of the model when applied to respiratory viruses, we sought to examine whether the genomic characteristics and model that was effective for respiratory diseases would be generalizable to non-respiratory viruses. Given that the model construction and evaluation was limited to respiratory viruses, an evaluation on other diseases or types of viruses may be confounded by the different tissue types. To mitigate possible confounders resulting from genome structure and affected tissue types, we focused on non-segmented ssRNA viruses of the families *Filoviridae* (negative-sense RNA viruses) and *Flaviviridae* (positive-sense RNA viruses) that cause hemorrhagic fevers. The hemorrhagic fever viruses were selected because they consist of a large enough set of viruses that are not associated with a specific tissue, and thus appear to be less likely to introduce bias in evaluation. Indeed, the model accurately predicted the incubation period of these viruses, including 3 types of Ebola viruses, Marburg virus, dengue virus, yellow fever virus, and tick-borne encephalitis virus (Fig. 4c, Spearman rho = 0.76, *p* value = 0.05).

## Discussion

The emergence of novel viruses that can cause pandemics remains a major threat to human health as compellingly demonstrated by the COVID-19 pandemic. A major challenge in dealing with such outbreaks is the initial lack of biological and clinical knowledge of the infectious agent, which can lead to potentially avoidable fatalities until the causative agent is thoroughly characterized. Therefore, to mitigate emerging outbreaks, rapid estimation of the incubation period of novel viruses is vital, in order to define the appropriate quarantine period and to estimate the rate of virus spread. Furthermore, we show here that the length of the incubation period in human viral diseases significantly correlates with the disease severity which further underlines the importance of the accurate prediction of the incubation time.

With recent advances in sequencing technology, genomic sequences of multiple isolates of novel viruses become available shortly after the virus emerges. Here, we comprehensively examined genomic features that could be predictive of the virus incubation times of human pathogenic ssRNA viruses and identified four family-generic features that consistently predict the incubation periods with high accuracy. Using these features, we developed a robust model that is predictive of incubation times for respiratory ssRNA viruses, the most common cause of viral pandemics [31]. Despite having been trained and evaluated on respiratory ssRNA viruses only, our model was found to be predictive also of the incubation periods of viruses that cause hemorrhagic fevers. Thus, the four genomic features that we identified as being family-generic allow for robust prediction of incubation periods for vastly different diseases caused by viruses that belong to different phyla [32]. Future advances based on this work can be expected to expand the model and feature search to additional sets of viruses and should comprehensively evaluate the effects of different confounders on the prediction, such as segmented genomes (for example, the influenza genome) and differences in the tissue tropism.

We also investigated the links between incubation periods of different disease-causing viruses and the disease severity and found that viruses with long incubation periods tend to cause severe disease. Although the relationship between incubation periods and disease severity has been assessed previously for specific viral diseases [6–8], to our knowledge, this connection has not been studied systematically across a large collection of human pathogenic viruses. This signal is robust across different viral families and disease types, including coronaviruses. To date, the study of virus incubation periods has been largely limited to human viruses. It remains to be explored whether the incubation periods of animal viruses correlate with those of human viruses. If there are robust correlations, these could provide additional avenues to investigate the effect of the incubation period on viral pathogenicity and infectivity in an evolutionary context, and perhaps, contribute to the development of early interventions for potential zoonotic viruses. Furthermore, such investigation could help with uncovering new coronaviruses with high pathogenic and zoonotic potential.

The underlying molecular and biological mechanisms of the dependencies between the family-generic genomic features, the incubation times, and disease severity remain to be directly and functionally investigated. One contributing factor could be a direct mechanistic connection between increased translation times in viruses with many genes and high GC content and longer incubation periods. Additionally, longer incubation periods are indicative of complex virus-host interactions that consequently present with more severe disease symptoms. This explanation is compatible with the observations in coronaviruses, whereby the highly virulent strains with long characteristic incubation periods encode several accessory proteins [21, 22] that are missing in viruses causing milder disease and have been implicated in virus-host interactions [23]. The domain content of some of these accessory proteins, indeed, seems to implicate them in interactions with the host immune system [24]. Another possible explanation is, simply, that a longer incubation period can lead to delayed medical intervention, so that by the time clinical symptoms appear, the medical intervention is less effective, and the disease presents as more severe. However, confirming or dispelling any of these hypotheses requires extensive virological experimentation.

## Conclusions

We demonstrated a robust association between virus incubation times and the severity of disease presentation and identified a set of viral genomic features that is highly predictive of incubation times. To our knowledge, this work is the first to demonstrate that incubation periods of respiratory ssRNA viruses can be accurately predicted by genome analysis alone. The model established through this work and the genomic features that were used for training can directly facilitate early and accurate estimation of the required quarantine time for future pandemics and help the responsible agencies set initial guidelines accordingly. Furthermore, these results have clear applications for controlling the spread of emergent ssRNA respiratory viruses, the most common cause of pandemics. Future work can expand this method to encompass additional virus families of interest and aid in mitigating the effect of potentially deadly zoonotic outbreaks.

## Methods

### Incubation period and severity assignment

The incubation time for each of the strains of each of the 41 viruses was collected from the literature (Additional file 1: Table S2). As incubation periods vary, where possible, the upper limit was used, and a consensus of reports was followed. The only exception to this is SARS-CoV-2. Although more data is needed to assess the incubation period of SARS-CoV-2, we set the duration to 14 days given the recommended quarantine times [2]. We note that there are small variations in reports of the incubation times, and the assigned values represent the best approximation. Changing the assigned incubation times within the range of reports maintains a similarly high performance of the trained model (Additional file 1: Figure S2).

The rationale for the selected incubation times for the 14 ssRNA respiratory viruses used in model construction and assessment was as follows:

a *229E-CoV (n = 25), HKU1-CoV (n = 39), NL63-CoV (n = 60), and OC43-CoV (n = 161).* For these coronaviruses, which are causative agents of common colds, a 3-day incubation period was assigned, following the majority of reports [33, 34].
b *MERS-CoV (n = 284)*. A 14-day incubation period was assigned to MERS-CoV, per previous reports [35].
c *SARS-CoV (n = 273).* The estimates show 13 days as an upper limit in the majority of reports [4, 36].
d *SARS-CoV-2 (n = 92)*. Although more data is needed to assess the incubation period of SARS-CoV-2, we set the duration to 14 days given the recommended quarantine times [2].
e *Measles virus (n = 213)*. There is a considerable range of reported incubation periods, with the majority of reports indicating 9–12 days and some reports going several days beyond that. Thus, we set the incubation period to 14 days [18].
f *Respiratory syncytial virus (RSV, n = 1595).* For RSV, the incubation period was set to 8 days, in accordance with the higher range of the majority of reports [37].
g *Parainfluenza (n = 43, 58, and 345 for parainfluenza 1, 2, and 3, respectively)*. The parainfluenza incubation period was consistently reported to be between 2 and 6 days and therefore was assigned the upper limit of 6 days [4].
h *Rhinovirus (n = 244)*. Per previous reports, a 4-day incubation period was assigned to rhinovirus [4].
i *Metapneumovirus (n = 162)*. Six days was assigned to human metapneumovirus, given the commonly reported range of 4–6 days [38].

We also assigned each of the 41 viruses with a binary severity annotation, of either severe or mild. Diseases with extreme immune responses, fevers, or other extreme symptoms were considered severe, along with diseases with high death rates. Diseases that cause mild respiratory symptoms or diseases that are otherwise benign were considered mild. In cases where either the Centers for Disease Control and Prevention (CDC) or the WHO explicitly described a disease as either severe or mild, that description was applied as the severity annotation. The disease presentations and severity determined, along with the rationale for the determined severity, are detailed in Additional file 1: Table S2.

Each of the 41 viruses was also classified by its symptoms and affected tissues, based on CDC and WHO descriptions, falling into one of these categories: central nervous system (CNS), fever, gastro, gastro/CNS, hemorrhagic fever, immune system, liver, skin, and swollen glands.

### Sequence datasets

Reference genome sequences and GenBank files were downloaded from the NCBI [39] for each virus (Additional file 1: Table S1, Additional file 2). For each virus, additional strains were downloaded from the NCBI and aligned using Mafft [40] v7.407 with default parameters, resulting in an alignment file for all strains belonging to each of the 14 viruses (Additional file 3). For each virus, all available strains were downloaded. Phylogenetic trees were generated for each virus based on the alignment using FastTree [41, 42] with the “-nt” parameter.

### Genomic features

The following genomic features with potential links to viral replication time and efficiency were evaluated:

a *Genome length*. The number of nucleotides in the reference genome sequence. Rationale: The length of the genome might correlate with virus reproduction time.
b *Number of genes*. The number of genes in the reference genome’s associated GenBank file. We verified for each virus that there were no undetected genes within its genome using MetaGeneMark [43] gene prediction software. Rationale: The number of genes might correlate with the total time spent on translation in the viral lifecycle and, thus, with the reproduction time.
c *Positive or negative strand RNA.* Whether the RNA virus is positive strand or negative strand. This was set to 1 if the virus has a positive strand genome and to 0 if it has a negative strand genome. Rationale: The positive- or negative-sense RNA might correlate with the time required to begin translation; negative ssRNA viruses require an additional stage to synthesize the positive-sense antigenome before translation, and accordingly, could correlate with the reproduction time.
d *Codon adaptation index (CAI)*. The CAI was used to analyze the codon usage bias of each virus in comparison to human. The CAI was calculated by concatenating all the coding sequences (CDS) in each virus reference genome GenBank file and using the Biopython [44, 45] software package (version 1.74) implementation, with the *CodonAdaptationIndex* class set to a reference human codon usage table [46]. Rationale: The codon adaptation index could correlate with translation efficiency and thus with the viral reproduction time.
e *GC content*. This was calculated for each reference genome using Biopython [44]. Rationale: GC content could correlate with translation times [20] and thus with the reproduction time.
f *Mutation rate.* Raw mutation rates were estimated per each virus genome alignment, without accounting for selection bias, by detecting the ancestral base for every base in the genome for every non-leaf node in the tree using maximum parsimony. Then, at each branch point, the transitions between both sides of the branch were counted, and the average count was then divided by the length of the genome for the final estimate. Rationale: The mutation rates could correlate with the reproduction time [47].
g *Average and variance of the changes in each position of the alignment.* The change in alignment position is defined as the number of different values observed in each position of the virus alignment, divided by the number of strains in the alignment. The average and variance of these values are used as features. Rationale: The position change mean and variance might correlate with the translation efficiency and regulation among different genomic regions and, thus, with the viral incubation period.

Features that rely on the multiple sequence alignment of different strains of the same virus were always normalized by the number of strains available, in order to avoid biases that could result from different strain counts per virus.

CpG islands were searched for in each reference genome using a Python implementation (https://github.com/lucasnell/TaJoCGI) with standard criteria [14]. However, none was found in any of the analyzed virus genomes.

### Evaluation of the specificity of the features for virus families

We sought to evaluate, for each feature, whether it is associated with the identity of the virus family which would be a potential confounder to the model. We hence searched for features whose variance within each virus family was not significantly smaller than its overall variance. To this end, each feature was evaluated using two methods.

The first method is a one-way analysis of variance (ANOVA). One-way ANOVA tests the null hypothesis that the means of the measurement variable are the same for the different categories of data, against the alternative hypothesis that they are not all the same. Hence, lower assigned *p* values signify that the null hypothesis is rejected and that different viral families have different population means with respect to each feature. We therefore consider features assigned with a *p* value greater than 0.05, for which we could accept the null hypothesis, and could not conclude that the feature mean was associated with the viral family. The ANOVA test was implemented in Python using the f_oneway function in the SciPy [48] package.

The ANOVA test assumes that the samples are independent, taken from normally distributed populations with equal standard deviations between the groups. These assumptions, which must be satisfied for the associated *p* value to be valid, are not guaranteed and are difficult to evaluate. We hence implemented a second, empirical test, which is not parametric and does not rely on any assumptions. This empirical test evaluates, for a given feature, if its variance within virus families is smaller than would be observed by random assignment of families to viruses. We reason that a feature which is associated with the virus family would have significantly smaller variance within the true family assignment than within a random family assignment. The null hypothesis is that the variance of the features within each family is similar to the variance across families, and the alternative hypothesis is that the variance of the features within each family is smaller than the variance across families. To perform the empirical test, the feature variance within each virus family is calculated and averaged. Next, the feature values are randomly permuted 1000 times and the same calculation is performed, to generate a null distribution. Let the number of times the variance of the permuted values is less than the variance of the real values be *X*. The *p* value is calculated as (*X* + 1)/(1000 + 1). Thus, a lower *p* value indicates that the feature’s within-family variance is smaller than our null expectation. We search for features with a *p* value greater than 0.05, for which we conclude that the variance within the actual families is not smaller than that within randomly assigned families. This evaluation does not necessarily indicate that the family-specific features are poor predictors, rather, that, with the data available, it would not be possible to discern whether the signal from these features is primarily driven by the variation between virus families.

### Elastic net model

The elastic net method [49] is a generalization of LASSO using Ridge regression shrinkage, where the naïve estimator $\hat{\beta\ }$ is a minimizer of the criterion *L*(*λ*_1_, *λ*_2_, *β*) by:

$$ \hat{\beta}={\mathrm{argmin}}_{\beta}\left(L\left({\lambda}_1,{\lambda}_2,\beta \right)\right)={\mathrm{argmin}}_{\beta}\left({\left\Vert y- X\beta \right\Vert}^2+{\lambda}_1{\left\Vert \beta \right\Vert}_1+{\lambda}_2{\left\Vert \beta \right\Vert}^2\right) $$

for any fixed, non-negative *λ*_1_, *λ*_2_. Elastic net was chosen because it has characteristics of both LASSO and Ridge regression, which are controlled by the penalties coefficients, thus outperforming other regularization and variable selection approaches [49].

The elastic net model was constructed in Python using the scikit-learn [50] ElasticNet function with default parameters. The features were standardized before training, with the same standardization parameters used in training applied to test data before prediction.

### Evaluating intervals for the predicted incubation periods

Given that there is no consensus as to how to define standard errors or confidence intervals for LASSO, Ridge, and elastic net estimates [28–30], we develop an empirical estimation of the lower and upper range of the incubation period using the elastic net model. To this end, we trained two models on the training data (viruses in the *Coronaviridae* family), with the first model trained on the lower estimates of the incubation period of coronaviruses and the second model trained on the highest reported estimate of the incubation periods.

### Evaluating the significance of assigned interval using permutation test

To evaluate the significance of the correlation between the predicted incubation intervals and the true intervals, we applied a permutation test. We calculated the average deviation of the predicted ranges from the true incubation ranges across all viruses in the test set, which is 1.7 days. We then shuffle the true intervals 1000 times, to generate a null distribution. Let the number of times the average deviation of the predicted range from the permuted range is less than or equal to the average deviation of the predicted range from the true range be *X*. The *p* value is calculated as (*X* + 1)/(1000 + 1).

## Supplementary Information

**Additional file 1: Figure S1.** The performance of models trained with family-generic features. **Figure S2.** Model performance for ranging incubation time assignment. **Table S1.** Virus and disease features for 41 collected viruses. **Table S2.** Family-generic features for the 14 viruses studied.

**Additional file 2.** GenBank file with the reference genomes used for each of the studied viruses.

**Additional file 3.** Accessions of the nucleotide sequences used for each of the 14 viruses studied. The sequence alignments are available through Zenodo (https://zenodo.org/record/4239675).

## Data Availability

Genome sequences and GenBank files were downloaded from the NCBI [39]. Virus genome sequences and alignments used for this analysis are provided through Zenodo. (https://zenodo.org/record/4239675), with the DOI: 10.5281/zenodo.4239675. The full accessions list is additionally provided in Additional File 3. The model, features, and accompanying code are available at https://github.com/noamaus/incubation-model.
